# A Monocentric Analysis of Implantable Ports in Cancer Treatment: Five-Year Efficacy and Safety Evaluation

**DOI:** 10.3390/cancers16162802

**Published:** 2024-08-09

**Authors:** Adel Abou-Mrad, Luigi Marano, Rodolfo J. Oviedo

**Affiliations:** 1 Centre Hospitalier Universitaire d’Orléans, 45100 Orléans, France; adel.abou-mrad@orange.fr; 2 Department of Medicine, Academy of Applied Medical and Social Sciences–AMiSNS: Akademia Medycznych I Spolecznych Nauk Stosowanych, 82-300 Elbląg, Poland; 3 Department of General Surgery and Surgical Oncology, “Saint Wojciech” Hospital, “Nicolaus Copernicus” Health Center, 80-462 Gdańsk, Poland; 4 Nacogdoches Medical Center, Nacogdoches, TX 75965, USA; roviedo3@central.uh.edu; 5 Department of Surgery, Tilman J. Fertitta Family College of Medicine, University of Houston, Houston, TX 75965, USA; 6 Department of Surgery, Sam Houston State University College of Osteopathic Medicine, Conroe, TX 77304, USA

**Keywords:** totally implantable vascular access devices (TIVADs), central venous catheters (CVCs), chemotherapy delivery, cancer patient care, surgical techniques, complication rates

## Abstract

**Simple Summary:**

This research investigates the effectiveness and safety of totally implantable vascular access devices (TIVADs) used for delivering treatments such as chemotherapy. Over a five-year period, 70 patients received TIVADs through a standardized surgical method under local anesthesia. The study found very low complication rates, with only two notable incidents: one device needed adjustment due to a flipped catheter, and one infection occurred after over three years of use. The procedural success rate was 100%, with devices typically lasting around 22 months, and some even up to five years. These findings suggest that TIVADs are a reliable and safe option for long-term venous access, significantly benefiting routine clinical practices.

**Abstract:**

Background: Daily clinical practice requires repeated and prolonged venous access for delivering chemotherapy, antibiotics, antivirals, parenteral nutrition, or blood transfusions. This study aimed to investigate the performance and the safety of totally implantable vascular access devices (TIVADs) over a 5-year follow-up period through a standardized well-trained surgical technique and patient management under local anesthesia. Methods: In a retrospective, observational, and monocentric study, 70 patients receiving POLYSITE^®^ TIVADs for chemotherapy were included. The safety endpoints focused on the rate of perioperative, short-term, and long-term complications. The performance endpoints included vein identification for device insertion and procedural success rate. Results: The study demonstrated no perioperative or short-term complications related to the TIVADs. One (1.4%) complication related to device manipulation was identified as catheter flipping, which led to catheter adjustment 56 days post-placement. Moreover, one (1.4%) infection due to usage conditions was observed, leading to TIVAD removal 3 years and 4 months post-surgery. Catheter placement occurred in cephalic veins (71.4%), subclavian veins (20%), and internal jugular veins (8.6%). The procedural success rate was 100%. Overall, the implantable ports typically remained in place for an average of 22.4 months. Conclusions: This study confirmed the TIVADs’ performance and safety, underscored by low complication rates compared to published data, thereby emphasizing its potential and compelling significance for enhancing routine clinical practice using a standardized well-trained surgical technique and patient management.

## 1. Introduction

Totally implantable vascular access devices (TIVADs) are long-term central venous access devices consisting of a chamber connected to an intravenous catheter, placed under the skin [1]. TIVADs were introduced in the early 1980s, presenting a promising option for long-term venous access [2,3]. These ports are the most commonly used central venous catheters (CVCs) for safe chemotherapy infusion, antibiotic administration, and blood sampling, avoiding repeated venipunctures and minimizing discomfort in patients, such as cancer patients who commonly require repeated central venous access. They improve the patient’s quality of life with a better cosmetic appearance, allowing for easier daily activities [1,4,5,6,7,8,9]. TIVADs can be essential for the management of several acute and chronic conditions, such as cystic fibrosis, congenital cardiac disease, or hemodialysis [10].

For TIVAD insertion, recent studies demonstrated an excellent technical success rate (99.3%) using the modified Seldinger technique under real-time ultrasound and fluoroscopy guidance, with low complication rates [5,9]. They are considered the preferred devices for systemic therapy in gynecological cancer patients and are effective and safe vascular access devices for breast cancer patients. The most commonly used insertion sites are the jugular vein or subclavian vein in the anterior chest, but recent studies showed that TIVAD insertion in the upper arm is also safe and widely used in breast cancer patients due to the low pneumothorax rate and better aesthetic appearance [2,11,12].

Several studies compared the safety and efficacy of TIVADs to other central venous catheters in cancer patients and reported that TIVADs showed higher levels of safety and efficacy, with optimal patient satisfaction [3,7,12,13]. Despite their high safety profile, their use can be associated with perioperative and long-term complications such as infection, venous thrombosis, and fluid extravasation [4,8,14]. The complication incidences are lower in comparison to external CVCs in adults and children. The risk of Central Line-Associated Bloodstream Infection (CLABSI) with TIVADs is lower compared with external CVCs (RR = 0.44; 95% CI, 0.31–0.62). Moreover, the rate of venous thromboembolism was 2.76% (95% CI, 2.24–3.28) in patients with TIVADs versus 4.86% (95% CI, 4.08–5.64) in PICC (Peripherally Inserted Central Catheter) recipients [15,16]. Perioperative complications include cardiac arrhythmia, accidental arterial injury, hemothorax, pneumothorax, and, rarely, air embolism [1]. In 81% of cancer patients, late device infections can lead to premature removal or replacement, with the risk of interruption or delay in treatment, which can generate stress and increase morbidities [3,4,8,15].

The incidence of associated sepsis in this population is estimated at around 0.5–10 per 1000 CVC-days [6]. Infection is the most frequent complication causing the implantable device’s premature port removal, with Staphylococcus and Candida infections being the most frequent findings. The infection occurs during use, especially with blood sampling and flushing [2]. The inserted device should, therefore, be handled by skilled staff. Antibiotic prophylaxis is advised for the insertion procedure or as a flush for the unused catheter [17].

The present study aimed to retrospectively evaluate the performance and safety of POLYSITE^®^ (Perouse Medical, Vygon Group, Paris, France) implantable ports in cancer patients over a 5-year follow-up period and evaluate the impact of procedure standardization on feasibility, success rate, and lowering of complications incidence. It assessed the rate of perioperative complications, as well as short- and long-term complications related to a standardized operation technique. The veins used and the subcutaneous port insertion sites were evaluated. The types of treatments administered via the medical devices were assessed, alongside the procedural success rate.

## 2. Materials and Methods

### 2.1. Study Design

This was a retrospective, observational, and monocentric descriptive study including 70 patients with POLYSITE^®^ implantable ports manufactured by Perouse Medical, Vygon Group. The safety objectives included the rate of perioperative complications, short-term and long-term complications, and the identification of new emergent risks. The performance endpoints were defined as the identification of the veins selected for catheter insertion and the rate of procedural success on the day of surgery.

Inclusion criteria: Adult patients from the Unit of Ambulatory Surgery Department (UASD) of the University Hospital Center La Source (Orléans, France), who underwent implantation procedures between 1 October 2016 and 31 December 2017, for chemotherapy. Data were collected from implantation until port removal.

Exclusion criteria: Patients’ refusal for data collection according to the Réglement Général de Protection des Données (RGPD) regulation applicable in France. Patients with unlisted device references in the study protocol. Patients operated on after 31 December 2017.

### 2.2. Patient Management and Surgical Technique 

Patients scheduled for TIVAD placement are seen on the surgeon’s consultation day, approximately one to two weeks before the scheduled surgery, unless immediate intervention is necessary. The surgery is then scheduled, typically on a designated operative day established weekly. The surgeon assumes responsibility for the anticipated number of patients, and a dedicated operating room (OR) is arranged accordingly. Patients are admitted alternatively to the UASD, and if possible, walk to the OR. 

Notably, no antibiotic prophylaxis was administered in this study. The placement of the TIVAD was performed using a surgical technique, with patients positioned on radiolucent wheeled stretchers. A solution of 150 mg of 7.5% Naropin^©^ (Fresenius Kabi, Lake Zurich, IL, USA) diluted with normal saline solution on a volume-to-volume basis, was injected into the deltopectoral groove site, both subcutaneously and intramuscularly, followed by administration at the pectoral implantation site. Subsequently, necessary surgical materials were prepared, and the TIVAD POLYSITE^®^ Perouse Medical, Vygon Group, was rinsed with normal saline solution.

The incision was made parallel to the deltopectoral groove, enabling access to and ligation of the cephalic vein on its brachial side. Next, a cutdown was performed, allowing for the insertion of the catheter into the vein and movement towards the superior vena cava. Radiologically guided placement occurred approximately one to two centimeters below the carina. Finally, the catheter was connected to the port positioned on the pectoral muscle.

To verify the device’s functionality, a “Huber” needle was used to assess blood reflux flow, followed by the injection of 30 to 50 mL of normal saline solution. The needle was subsequently removed under positive continuous pressure, and closure of the subcutaneous tissue and skin was achieved using resorbable sutures. Lastly, a final radiological X-ray assessment was performed to confirm proper TIVAD placement.

Patients were then able to return to the UASD, and if clinically asymptomatic, they were discharged following two hours of clinical observation.

### 2.3. Ethical Considerations and Patient Consent

This retrospective study was conducted in accordance with the ethical principles of the Declaration of Helsinki and regional or national regulations applicable in France. No Ethics Committee review was required.

This study complies with the General Data Protection Regulation (GDPR) and the Methodology of Reference MR-004 developed by CNIL (French Data Protection Agency). The principles outlined in ISO 14155:2020 [18] were followed as far as possible, considering the nature of this study (retrospective collection of data).

An information letter and consent form for the patients who undergo TIVAD implantation were set up. This study was registered in a public database under the registration number CNIL 2224317 V0 (Website: https://clinicaltrials.gov/study/NCT05519787?intr=polysite%20&rank=2&tab=table, accessed on 30 July 2024).

### 2.4. Statistical Analyses

All patients who met the eligibility criteria were included in the study population. A descriptive analysis with clinical characteristics was performed at the index date. Exploratory analyses were conducted for the global population. The study objectives were addressed using descriptive statistics only, as no hypotheses were tested. Statistical calculations were carried out using R software^®^ (version 4.4.0).

To determine the minimum sample size required to achieve sufficient statistical power for detecting a meaningful effect size, we conducted a power analysis based on the specific endpoints and anticipated variability within the data. The sample size calculation utilized Cohen’s effect size (d) set at 0.3, representing a small to medium effect size in medical research.

The analysis was performed with a significance level (alpha) of 0.05 and a desired power of 0.80, employing G*Power software (version 3.1.9.7) [19]. These parameters were chosen to minimize the likelihood of Type I and Type II errors, thereby ensuring the reliability and validity of the study findings. Based on these criteria, the required sample size was calculated to be 71 patients. The study included 70 patients, closely meeting the calculated requirement, which ensures that the results are robust and generalizable.

## 3. Results

### 3.1. Patient Characteristics

Seventy patients (22 (31.4%) males, 48 (68.6%) females) who received a POLYSITE^®^ TIVAD were enrolled. The mean age of the entire cohort was 62.2 years (SD = 12.25), with a female mean age of 60.9 years (SD = 12.02) and a male mean age of 65 years (SD = 12.55) (Table 1).

**Figure 1:**
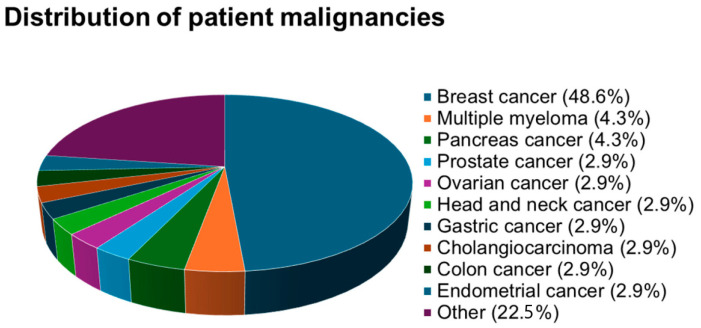
Distribution of patient malignancies.

The predominant diagnosis at the time of study inclusion was breast cancer with 34 cases (48.6%), multiple myeloma, and pancreatic cancer, each diagnosed in three patients (4.3%). Additionally, cholangiocarcinoma, colon cancer, endometrial cancer, gastric cancer, head and neck cancer, ovarian cancer, and prostate cancer, were each observed in two patients (2.9%). The comprehensive overview of various malignancies within the studied population is presented in Figure 1.

### 3.2. Device Characteristics

A total of 70 medical devices were assessed. The predominant device range used by the physician was 4008 ISP (peel-away sheath introducer), comprising 63 (90%) devices, followed by 4 (5.7%) devices of 3007 ISP and 3 (4.3%) of 4008 ECHO (echogenic). All inserted TIVAD ranges consist of a titanium reservoir and a polyoxymethylene (POM) outer casing and a silicon catheter. A detailed overview of the different devices’ characteristics is shown in the following Table 2.

### 3.3. TIVAD Placement

Catheterization (Table 3) of the right cephalic vein was the most frequent procedure observed in 36 (51.4%) cases. In 14 (20%) cases the left cephalic vein and in 12 (17.1%) cases the right subclavian vein were used. In six (8.6%) and two (2.9%) cases, the catheter was placed in the right jugular and left subclavian vein, respectively. The corresponding catheter ports (Table 4) were placed in the right pectoral muscle in 54 (77.1%) cases and the left pectoral muscle in 16 (22.9%) cases.

### 3.4. Procedural Success

**Figure 2:**
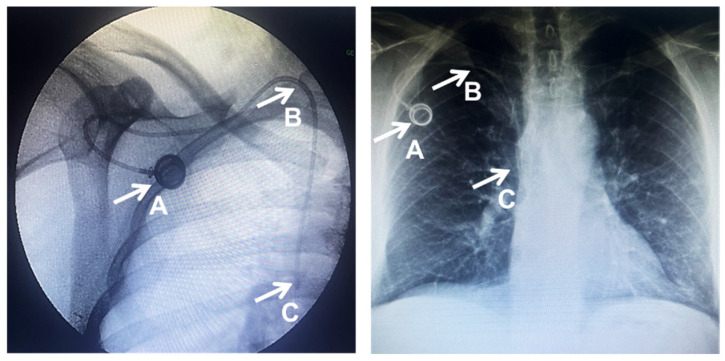
Radiological TIVAD placement confirmation. A. Port, B. catheter, C. catheter tip.

The procedural success was defined as proper device placement, successful blood reflux and serum injection tests, and radiological confirmation of the adequate position of the catheter’s tip. The procedural outcome parameters were assessed in all cases, resulting in a 100% rate of success and successful radiological control of the port, (Figure 2, A) where the catheter (Figure 2, B) and the catheter tip’s location (Figure 2, C) are at one or two centimeters below the carina projection.

Seventy TIVADs were placed during 9 dedicated days, with an average of 7.7 catheters inserted per day. The insertion procedure duration, also called intervention time, was also reported. In all the cases (100%), the intervention time was 10 min.

### 3.5. Catheter Removal throughout the Follow-Up Period

During the follow-up period, 46 (65.7%) patients maintained the initially inserted TIVAD (Table 5), with a mean follow-up period of 973.1 days (SD = 678, 2.67 years). The minimal impact on patient comfort and insignificant adverse effects allowed for prolonged catheter retention, with some patients keeping it for potential venous access. Conversely, TIVADs were surgically removed from 24 (34.3%) patients. Specifically, TIVAD removal occurred in 23 (32.9%) patients, with a mean of 659.5 (SD = 449.6) catheter days upon completion of therapeutic intervention. An average follow-up period of 1759.9 days (SD = 115.7) was reported in these 23 patients. In one case (1.4%), device removal was necessary due to the observation of an infection after 1198 days (3.32 years), and the patient was followed up for 16 days after the catheter’s removal.

### 3.6. Complications

During the follow-up period of this study, no minor or major medical device-related complications were reported. However, two long-term adverse events occurred in two patients: one (1.4%) case of catheter migration and flipping/rotation of the port in one patient, and one (1.4%) infection with Staphylococcus epidermidis in the other patient. Importantly, these complications were related to device manipulation/use by the hospital staff. The catheter migration and port flipping/rotation occurred 56 days after the insertion, and the catheter was restored to its original position. The infection observed in one patient occurred 1198 days (3.28 years) after the TIVAD’s insertion, and prompt treatment within an appropriate timeframe successfully resolved the infection.

## 4. Discussion

TIVADs are used in chemotherapy, parenteral nutrition, frequent need for blood transfusions, long-term antibiotic therapy, and other conditions requiring chronic central intravenous access. Alongside PICCs, TIVADs are the most frequently employed CVCs for chemotherapy administration in gynecological cancers. The use of TIVADs for chemotherapy is a common adjunct aiming to enhance the quality of life for patients by allowing them to maintain their daily activities and achieve better aesthetic outcomes. Additionally, their use is associated with fewer complications compared to PICCs, particularly a lower risk of thrombosis [11].

In our study, TIVADs were utilized to deliver chemotherapy primarily to breast cancer patients (48.6%). All catheters used in this study were made of silicone rather than polyurethane. Historically, silicone has been used for long-term venous catheters since their introduction in 1973. Wildgruber et al. demonstrated that polyurethane catheters were more susceptible to catheter-related infections and exhibited higher thrombogenicity than silicone catheters, which had decreased mechanical stability [20]. Moreover, Busch et al. reported that silicone catheters were more prone to material failure and mechanical complications, while polyurethane catheters were more associated with venous thrombosis [21]. Another study by Alzahrani et al. assessed the impact of catheter material on port removal and showed that silicone-based catheters were less vulnerable to rupture than polyurethane-based catheters. In fact, fibrin formation around polyurethane catheters could lead to catheter fixation to the blood, which can cause catheter fracture [22].

TIVADs can be placed by an open surgical approach involving a cutaneous incision or a percutaneous approach where the needle is inserted blindly or under ultrasound guidance [22]. In this study, the cephalic vein was the most used insertion vein (71.4%) and in the majority of the studied breast cancer patients. The cephalic vein is surgically preferred, while the internal jugular vein is better approached percutaneously.

In our study, the surgical cutdown technique was performed rather than the Seldinger percutaneous technique. Cephalic vein surgical cutdown is the quickest and safest technique with a good success rate. In the case of failure to locate or use the vein or its inconsistency, the subclavian and internal jugular veins are alternatives. Although those locations may increase the risk of pneumothorax, hematoma, arteriovenous fistula, and residual pain, ultrasound localization of the central vein helps with reducing the incidence of complications. The choice of insertion technique was made to avoid these complications, which can be lethal and more associated with the Seldinger technique, especially in patients with a compromised quality of life due to their cancer [23]. In breast cancer patients, TIVADs are traditionally placed contralateral to the disease to minimize potential complications associated with ipsilateral port placement [24].

Managing surgical factors, including the proficiency of the healthcare professional performing TIVAD insertion, can reduce complication rates. In our study, the surgeon had extensive training and experience in catheter insertion, performing multiple interventions per day with a short intervention time, which may contribute to a lower occurrence of complications. Standardized surgical techniques and patient management enhance the patients’ tolerance of the surgical procedure under local anesthesia, their comfort during implantation and treatment, and overall satisfaction. A well-executed and reproducible technique helps with shortening the operative time and may prevent the incidence of infection.

In our study, there were no perioperative infections, and only one long-term infection occurred, which was attributed to usage conditions rather than the device itself. This low incidence of infections can largely be attributed to stringent infection prevention protocols. Specifically, for TIVAD implantation at our hospital, no perioperative antibiotics were administered, a practice that aligns with our findings of no early infections. This approach is consistent with the recent literature, where infection rates vary widely from 1.6% to 50% [25,26,27,28,29,30]. The standardized and well-executed surgical technique used for TIVAD insertion, along with meticulous intraoperative and postoperative care, played a crucial role in minimizing infection risks [31,32]. The decision to refrain from routine antibiotic prophylaxis was offset by strict adherence to aseptic techniques, ensuring a sterile environment and careful handling of the devices [17,32]. A key component of this infection control strategy was the use of silicone catheters, which are associated with lower thrombogenicity, and a reduced risk of bacterial colonization compared to other materials like polyurethane [20,21]. This choice, along with diligent management and handling of TIVADs by trained personnel, especially during high-risk procedures such as blood sampling and flushing, likely contributed to the low infection incidence observed.

These findings highlight the importance of a comprehensive infection prevention strategy that includes both procedural and material considerations, thereby minimizing the risk of complications associated with TIVAD use [32,33].

Interestingly, there were no minor or major complications reported, such as hematoma or pneumothorax. The insertion success rate was 100%. The device proved to be practical and easy to place, with no complications or complaints reported. Overall, no medical device-related complications were observed. The main reason for device withdrawal was the completion of treatment. 

While our study demonstrated a low overall complication rate associated with TIVADs, the potential for thrombotic events, including thrombosis and subsequent pulmonary embolism (PE), warrants attention [1,15,16]. Thrombotic complications, though rare, can arise from endothelial injury during catheter placement, activation of coagulation pathways, or stasis of blood flow around the catheter. These conditions may occasionally lead to thrombi formation, which could embolize and result in PE, a critical clinical concern [34].

The comparative risk of venous thromboembolism associated with TIVADs and PICCs has been extensively studied. A comprehensive meta-analysis, which included 22 studies encompassing 11,940 patients, demonstrated that the risk of thromboembolism was significantly lower in patients with TIVADs than in those with PICCs. Specifically, the odds ratio (OR) was found to be 0.38 (95% CI: 0.25–0.58), indicating a substantially lower risk with TIVADs [35]. This reduced risk is attributed to the fully implanted nature of TIVADs, which limits the exposure to external contaminants and reduces the likelihood of infection, a significant risk factor for thrombosis [35,36].

Prophylactic measures, such as meticulous patient selection and the administration of anticoagulant therapy for high-risk individuals, are essential in mitigating the risk of thrombotic events [32,33]. Additionally, the use of advanced imaging techniques during catheter insertion can enhance procedural accuracy and minimize endothelial trauma, thereby further reducing the likelihood of thrombus formation [37,38].

In our study, we observed no cases of thrombosis leading to PE, which is consistent with the established safety profile of TIVADs. However, clinicians should maintain a high level of awareness for symptoms indicative of thrombosis since early detection and intervention are critical in preventing severe complications [39]. Although the incidence of thrombotic events with TIVADs is relatively low, ongoing monitoring and the implementation of appropriate preventive strategies are essential for ensuring patient safety [32,33,38]. This is particularly important for cancer patients, who are inherently at higher risk due to their underlying conditions and the therapeutic regimens they undergo [37].

While the total number of enrolled patients in this study may initially appear limited, it is crucial to highlight that the required sample size was meticulously determined through rigorous statistical calculations and strictly adhered to throughout the study. This adherence to a precisely calculated sample size significantly enhances the methodological rigor and robustness of our findings. Such an approach ensures the reliability and generalizability of our results, thereby reinforcing the study’s potential to offer valuable insights into the performance and safety of TIVADs. Consequently, the seemingly small sample size should be regarded as evidence of the careful planning and statistical precision that support this research, rather than as a limitation.

However, it is also important to acknowledge that recruiting a larger sample size could have increased the statistical power, provided more robust data analysis, and potentially offered deeper insights into the effectiveness of the surgical technique. Furthermore, the inclusion of patients with different venous access sources introduces heterogeneity into the data analyzed and presented. This variability may affect the uniformity and comparability of the results, complicating the interpretation of the findings. Therefore, the heterogeneity in venous access sources must be considered when evaluating the study’s conclusions and their applicability to more homogeneous patient populations.

Future research should consider planning a multicentric study with a larger, more homogeneous patient cohort to compare how patient management and surgical technique choices may prevent complications.

## 5. Conclusions

A well-trained surgeon and surgical team, a dedicated operative time, a standardized surgical technique, and a well-managed protocol are crucial elements for ensuring a well-tolerated and comfortable surgical procedure under local anesthesia, preventing the incidence of general anesthetic complications and particularly perioperative infections.

In summary, the use of TIVADs has been demonstrated to be safe, reproducible, and well tolerated by patients. Given the growing demand for long-term chemotherapy regimens and the increasing emphasis on improving patients’ quality of life, there is likely to be a significant rise in the need for TIVADs in the future. Nevertheless, additional prospective studies or more inclusive cohort studies are necessary to validate and confirm these findings.

## Figures and Tables

**Table 1:** Patient characteristics.

	Total		Male		Female	
Population Age Groups	n	%	n	%	n	%
<50 years	14	20.0	3	13.6%	11	22.9%
50–70 years	41	58.6	13	59.1%	28	58.3%
>70 years	15	21.4	6	27.3%	9	18.8%
TOTAL	70		22	31.4	48	68.6
Age (years)						
Mean (SD)	62.2 (12.25)		65.0 (12.55)		60.9 (12.02)	
Min–Max	28.0; 88.0		28.0; 84.0		34.0; 88.0	

**Table 2:** Device characteristics.

Device Range	Implantable Port Features		Catheter Features					Studied Devices	
	Prim. vol. (mL)	Material	Diameter (mm)		Material	French Size	Catheter Length (cm)	Number	%
			Outer	Inner					
3007 ISP	0.35	Titanium–Polyoxymethylene (POM)	2.16	1.02	Silicone	6.5	60	4	5.7
4008 ECHO	0.6		2.4	1.2		7.2	60	3	4.3
4008 ISP	0.6		2.4	1.2		7.2	60	63	90
TOTAL								70	

**Table 3:** Catheter placement.

Selected Vein	Number of Cases	%
Left cephalic	14	20
Right cephalic	36	51.4
Left subclavian	2	2.9
Right subclavian	12	17.1
Right jugular	6	8.6
TOTAL	70	

**Table 4:** Port placement.

Port Placement	Number of Cases	%
Left pectoral muscle	16	22.9
Right Pectoral muscle	54	77.1
TOTAL	70	

**Table 5:** Overview of catheter status and catheter days.

Catheter Status			Follow-Up Period (Days)			Catheter Days (Days upon Removal)		
			Mean (SD)	Min	Max	Mean (SD)	Min	Max
Removal								
Reason	n	%						
End of therapy	23	32.9	1759.9 (115.7)	1436	1890	659.5 (449.6)	211	1865
Infection	1	1.4	NA	NA	1214	1198		
TOTAL	24	34.3						
No removal								
	n	%						
TOTAL	46	65.7	973.1 (678)	28	1908	NA		

## Data Availability

The data presented in this study are available on request from the corresponding author due to confidentiality restrictions involving patient information.
